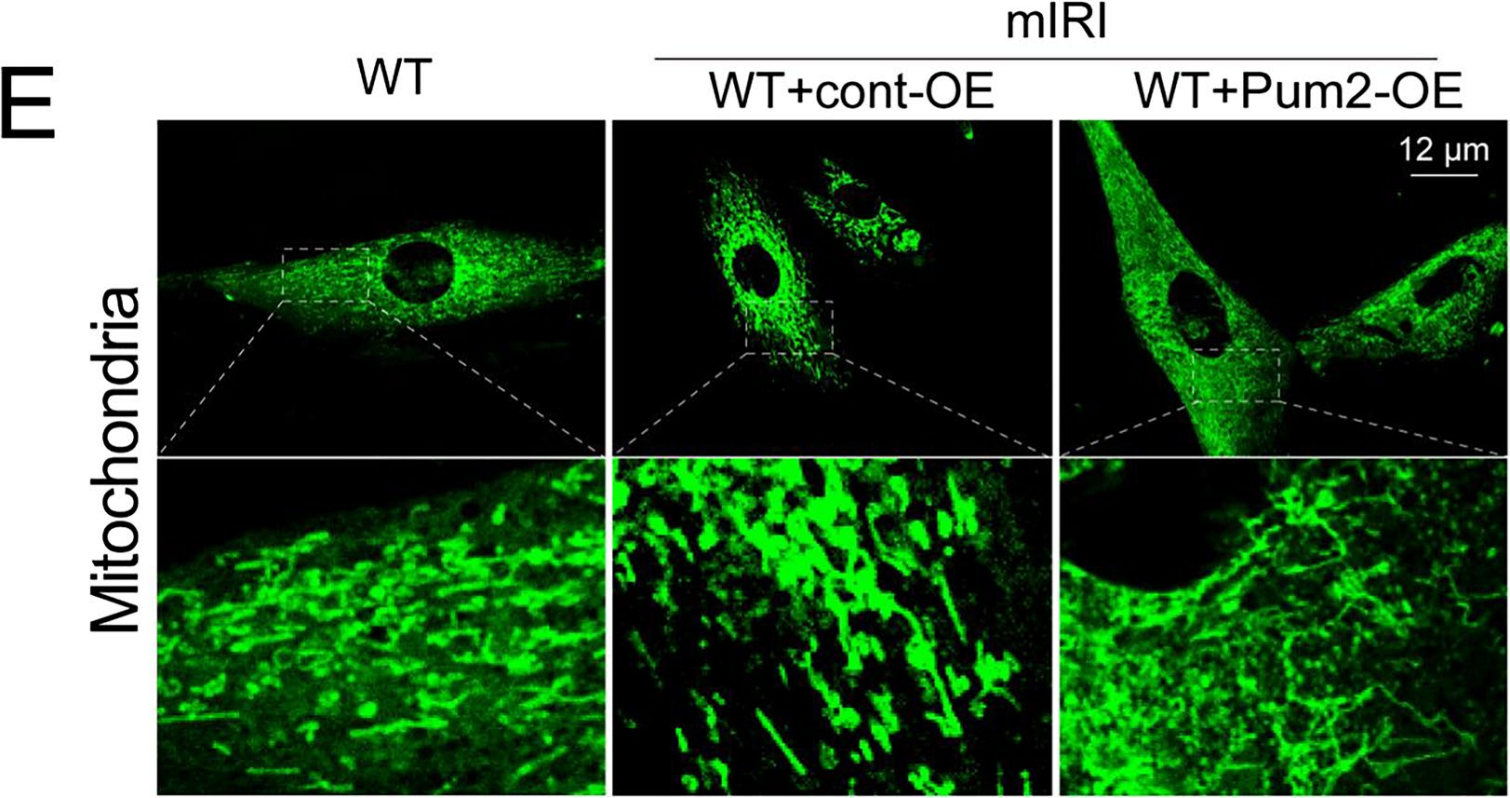# Correction to: Pum2-Mff axis fine-tunes mitochondrial quality control in acute ischemic kidney injury

**DOI:** 10.1007/s10565-024-09886-1

**Published:** 2024-06-13

**Authors:** Jin Wang, Pingjun Zhu, Sam Toan, Ruibing Li, Jun Ren, Hao Zhou

**Affiliations:** 1 Medical School of Chinese PLA, Chinese PLA General Hospital, Beijing, China; 2 Department of Chemical Engineering, University of Minnesota-Duluth, Duluth, MN 55812 USA; 3 Center for Cardiovascular Research and Alternative Medicine, University of Wyoming, Laramie, WY 82071 USA

**Correction to: Cell Biol Toxicol (2020) 36:365–378**

10.1007/s10565-020-09513-9

After the publication of this article, the authors would like to acknowledge an error in Figure 4E. The high-power field of the mIRI+WT+cont-OE group does not match the low-power field. We have included the corrected image of the high-power field of the mIRI+WT+cont-OE group as shown below.